# MiR‐5683 suppresses glycolysis and proliferation through targeting pyruvate dehydrogenase kinase 4 in gastric cancer

**DOI:** 10.1002/cam4.3344

**Published:** 2020-08-11

**Authors:** Yongchang Miao, Qing Li, Guangli Sun, Lu Wang, Diancai Zhang, Hao Xu, Zekuan Xu

**Affiliations:** ^1^ Department of General Surgery The First Affiliated Hospital of Nanjing Medical University Nanjing China; ^2^ Department of General Surgery The Second People's Hospital of Lianyungang Lianyungang China; ^3^ Jiangsu Key Lab of Cancer Biomarkers, Prevention and Treatment Jiangsu Collaborative Innovation Center For Cancer Personalized Medicine School of Public Health Nanjing Medical University Nanjing China; ^4^ School of Medicine Southeast University Nanjing China

**Keywords:** gastric cancer, glycolysis, miR‐5683, proliferation, pyruvate dehydrogenase kinase 4

## Abstract

Gastric cancer (GC) is one of the most deadly malignancies at global scale, and is particularly common in eastern Asia. MicroRNA‐5683 (miR‐5683) was confirmed to be downregulated in GC by analyzing data from the Cancer Genome Atlas. We packaged miR‐5683‐mimics and miR‐5683‐inhibitors into lentivirus vectors and transfected them into GC cells. MiR‐5683 expression and possible target genes were detected by employing quantitative real‐time polymerase chain reaction. In vitro, cell proliferation and apoptosis were analyzed using CCK‐8, colony formation assay, and flow cytometric assay. We verified the direct interaction between miR‐5683 and the possible downstream target gene pyruvate dehydrogenase kinase 4 (PDK4) through luciferase reporter assay. The role of miR‐5683 in vivo was explored by injecting stably transfected GC cells subcutaneously into nude mice. Here we show that miR‐5683 was downregulated in GC and the decreased level of miR‐5683 enhances GC cell proliferation and impairs apoptosis. Tumor oncogene PDK4, which is associated with GC overall survival and disease‐free survival, has been identified as the target gene of miR‐5683. Besides, we demonstrate that the inhibition of miR‐5683 promotes glycolysis by upregulating the PDK4 expression, thus leading to GC progression. Our study determines that miR‐5683 represses GC glycolysis and progression through targeting PDK4. MiR‐5683 overexpression may thus become a new treatment strategy for GC.

## Graphical abstract

MiR‐5683 expression is downregulated in gastric cancer (GC) tissues and cells, and miR‐5683 could suppress the proliferation and induce the apoptosis of GC. MiR‐5683 inhibits malignancy and glycolysis via suppression of PDK4 expression in GC.

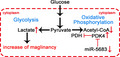

## 1 INTRODUCTION

Gastric cancer (GC) is a highly prevalent lethal malignancy at global scale, and is especially common in east part of Asia.^1^, ^2^ Despite dramatic advances in diagnosing and treating GC, 5‐year survival rate of patients who suffered from GC remains low.^3^, ^4^, ^5^, ^6^ As a result, the molecular mechanisms regulating GC progression require further studies.

MicroRNAs are noncoding and endogenously expressed RNAs, and typically 20‐24 nucleotides long.^7^, ^8^ Aberrant miRNA expression has been identified in many cancers and linked to diverse genes and signal pathways, and proved to influence tumor proliferation, viability, and metastasis. There is also evidence that miRNAs perform both oncogenic and anti‐oncogenic functions.^9^ MiR‐5683 was reported to promote proliferation and the migratory abilities of colon adenocarcinoma cells.^10^ Nevertheless, the possible functions of miR‐5683 in malignancies, especially in GC, are still mostly blank.

To meet the requirements of rapid growth of cancer cells, malignancies go through metabolic reprogramming to enable cancer cells to primarily utilize glucose for energy generation. This phenomenon was referred to as Warburg effect.^11^, ^12^ This process produces not only ATP, but also produces glycolytic intermediates that are required for rapid proliferation. Although it is widely known that the Warburg effect occurs in GC, the potential mechanism contributing to aerobic glycolysis remains to be uncovered in GC. Recently, miRNAs have been reported to play vital role in the regulation of glycolysis.^13^, ^14^, ^15^ However, in the field of GC, the effects of miRNAs on glycolysis need to be investigated further.

Herein, we assessed the functions of miR‐5683 in GC and show that miR‐5683 downregulation is a major factor in the progression of GC, a finding with significant implications for the development of new GC therapies.

## 2 MATERIALS AND METHODS

### 2.1 Tissue samples

A total of 70 patients who underwent radical resection for GC at the First Affiliated Hospital of Nanjing Medical University were enrolled in this study. Primary GC samples and matched paracancerous tissue were sent to the laboratory within 30 minutes of removal. No special treatment for GC was administered before surgery. All patients provided written informed consent and all procedures were approved by the Ethics Committee of the First Affiliated Hospital of Nanjing Medical University.

### 2.2 Cell culture

Gastric cancer cell lines and GES‐1 were provided by the cell bank of the Chinese Science Academy. These cells were cultured at 37°C with 5% CO_2_ in Roswell Park Memorial Institute (RPMI)‐1640 with 10% fetal bovine serum (FBS) and 1% antibiotics.

### 2.3 Quantitative real‐time polymerase chain reaction (qRT‐PCR)

Total RNA isolation was employed with TRIzol (Invitrogen) and cDNA was reverse transcribed using PrimeScriptRT reagent with a New Poly(A) Tailing Kit (Takara). We performed PCR amplification by employing a 7900 Real‐time PCR System (Applied Biosystems). We normalized miR‐5683 expression relative to U6. PDK1, PDK2, PDK3, and PDK4 expressions were normalized to β‐actin. The primers were as follows: miR‐5683 forward, 5′‐TACA GATGCAGATTCTCTGACTTC‐3′; Universal, 5′‐GCGAGCACAGAATTAATACG AC‐3′; U6 forward, 5′‐CTCGCTTCGGCAGCACA‐3′; U6 reverse, 5′‐AACGCTT CACGAATTTGCGT‐3′; PDK1 forward, 5′‐CCGCTCTCCATGAAGCAGTT‐3′; PDK1 reverse, 5′‐TGAACGGATGGTGTCCTGAG‐3′; PDK2 forward, 5′‐ATGG CAGTCCTCCTCTCTGA‐3′; PDK2 reverse, 5′‐CACCCACCCTCTCCCTAACA‐3′; PDK3 forward, 5′‐CGCTCTCCATCAAACA ATTCCT‐3′; PDK3 reverse, 5′‐CCACT GAAGGGCGGTTAAGTA‐3′; PDK4 forward, 5′‐GGAGCATTTCTCGCGCTACA‐3′; PDK4 reverse, 5′‐ACAGGCAATTCTTGTCGCAAA‐3′; β‐actin forward, 5′‐GCATCGTCACCAACTGGGAC‐3′; β‐actin reverse, 5′‐ACCTGGCCGTCAGGC AGCTC‐3′.

### 2.4 Lentivirus construction and infection

Lentiviral vectors with the PDK4 DNA sequence (LV‐PDK4), PDK4 siRNA hairpin sequence (PDK4 shRNA), miR‐5683‐mimics and miR‐5683‐inhibitor were provided by GenePharma. The target sequence of PDK4 shRNA was 5′‐ACTGCAACGTCTCTGAGGTG‐3′. All transfections followed manufacturer's instructions. Stable cells lines were selected using puromycin (Sigma) with a concentration of 5 μg/mL.

### 2.5 Colony formation assay

We placed 500 stably transfected GC cells into each well of 6‐well plates followed by culture for 3 weeks in RPMI‐1640 medium containing 10% FBS. Colonies were counted, photographed, and analyzed after being stained with crystal violet. Each procedure was replicated three times.

### 2.6 Cell counting assay

Viable cells grown in 96‐well plates were monitored using Cell Counting Kit‐8 (CCK‐8) (Dojindo) assays every 24 hours for 5 days according to the instructions.

### 2.7 Flow cytometry

To carry out cell cycle assay, cells were gathered and then centrifuged at 1500 rpm for 3 minutes. We rinsed the cells using PBS and fixed them with 75% ethanol through the night. Before detection, cells were rinsed using PBS two times and incubated with RNase. We eventually stained the cells with propidium iodide solution at room temperature. The staining lasted 15 minutes. To perform the apoptosis assay, collected transfected cells were rinsed and then resuspended in ice‐cold PBS. This was followed by staining with PI and Annexin V‐FITC for 15 minutes according to the instructions. Analysis was carried out using a FACScan flow cytometer.

### 2.8 Protein extraction and Western blot

We lysed GC cells using the Radio Immunoprecipitation Assay buffer (BioRad). The lysate was separated via SDS‐PAGE, with the obtained proteins being transferred to polyvinylidene (PVDF) membranes (Millipore). We blocked membranes with 5% skimmed milk for 2 hours at 37°C, and then incubated at 4°C overnight with the following specific primary antibodies: PDK4 (1/1000, ab89295; Abcam), c‐E1α (1/1000, ab168379; Abcam), phospho‐Pyruvate Dehydrogenase (PDH)‐E1α (Serine 293; 1/1000, ab177461; Abcam), and GAPDH (1/1000, ab181602; Abcam). We washed the membranes with Tris Buffered Saline with Tween 20 (TBST), and incubated them with HRP‐conjugated AffiniPure goat anti‐rabbit (1/5000, SA00001‐2; Proteintech) or anti‐mouse IgG (H + L) (1/5000, SA00001‐1; Proteintech) at 37°C for 2 hours. The PVDF membranes were washed three times with TBST buffer and then observed with an electrochemiluminescence (ECL) detection system. As an internal control, we selected GAPDH.

### 2.9 Luciferase reporter assay

Wild‐type (wt) and mutated (mut) 3'‐UTR sequences of PDK4 or PDK2 mRNA (Shenggong) were inserted into the pGL3 luciferase reporter vector (Promega) to yield the pGL3‐MUT‐PDK4, pGL3‐MUT‐PDK2, pGL3‐WT‐PDK4, and pGL3‐WT‐PDK2 3'‐UTR reporter plasmids. GC cells stably transfected with miR‐5683 or miR‐NC were placed with above 3'‐UTR reporter plasmids in 24‐well plates (5 × 10^5^ cells/well). As a control, we transfected GC cells with the Renilla luciferase expression plasmid (0.01 μg). Activities of Firefly luciferase and Renilla luciferase were detected after transfection for 36 hours as recommended by the manufacturer.

### 2.10 Oxygen consumption rate and extracellular acidification rate

We estimated oxygen consumption rate (OCR) and extracellular acidification rate (ECAR) based on the manufacturer's protocol, as previously published.^14^ Glycolytic capacity and cellular mitochondrial function were detected using a Seahorse XF24 analyzer (Seahorse Biosciences). Briefly, 2 × 10^5^ cells were seeded in Seahorse plates, incubated overnight, and washed in Seahorse buffer. Then, 175 μL of Seahorse buffer containing 25 μL each of 1 μmol/L oligomycin, 1 μmol/L FCCP, and 1 μmol/L rotenone was added to determine the OCR. To measure the ECAR, 175 μL of Seahorse buffer and 25 μL each of 10 mmol/L glucose, 1 μmol/L oligomycin, and 100 mmol/L 2‐DG were automatically injected into the analyzer.

### 2.11 Immunohistochemistry

Pyruvate dehydrogenase kinase 4 expression levels were estimated via immunohistochemistry (IHC) staining. Sections (3‐µm thickness) were prepared from deparaffinized tissue specimens and subcutaneous xenograft models samples, and incubated with the PDK4‐specific primary detection antibody through the night at the temperature of 4°C. In the next step, slices were rinsed three times with PBS. We incubated the slices with goat anti‐rabbit IgG for 30 minutes. Finally, the color reagent 3, 3′‐diaminobenzidine (DAB) was used for staining the sections. The intensity of IHC staining was scored as 0 (negative), 1 (weak), 2 (medium), and 3 (strong). The percentage of positive cells in the whole tissue slice was divided into five grades: 0 (≤10%), 1 (10%‐25%), 2 (26%‐50%), 3 (51%‐75%), and 4 (>75%). Intensity score and positive rate score were then multiplied to calculate the overall score.

### 2.12 Xenograft tumor model

Establishment of xenograft tumor model with nude mice was approved by Nanjing Medical University Ethics Committee. We obtained female nude mice (aged 4 weeks) from Vitalriver. They were kept free of specific pathogens. Mice were subcutaneously (bilateral flank) injected with stably transfected cell lines. Every 4 days the tumors were measured using calipers. Volumes of the tumors were calculated with the formula: volume = (width^2^ × length)/2. After 20 days, euthanized mice had their tumors removed.

### 2.13 Statistical analysis

SPSS (Version 22.0) was used for analysis. Data were expressed as mean ± standard deviation (SD). Pearson chi‐squared tests and Student's *t* tests compared clinicopathological findings. To test for significant differences across control and treatment groups, we applied analysis of variance (ANOVA). *P* < .05 was defined as statistically significant.

## 3 RESULTS

### 3.1 MiR‐5683 expression is downregulated in GC tissues and cells

**FIGURE 1:**
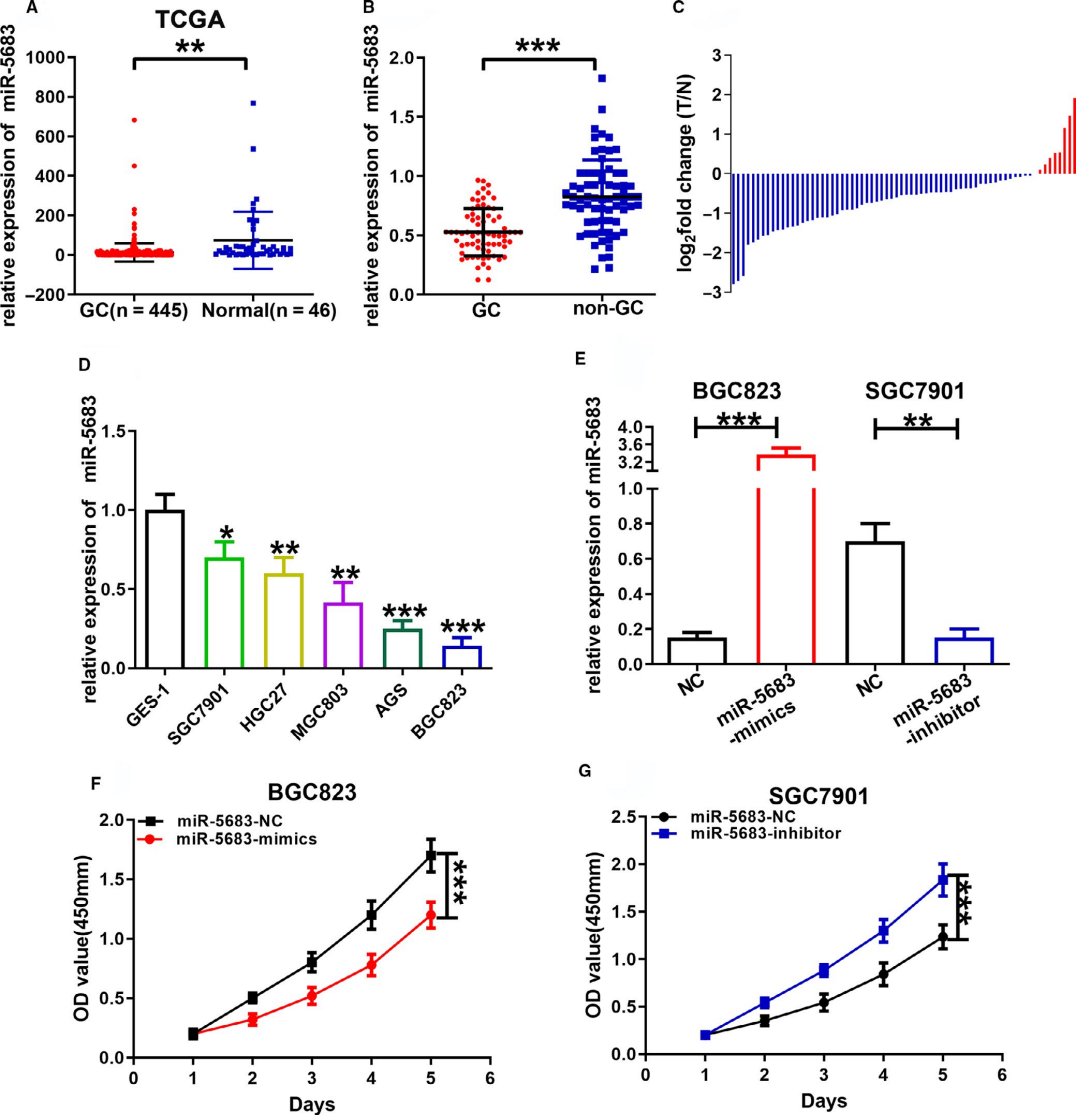
MiR‐5683 was downregulated in gastric cancer (GC) tissues and cells. A, MiR‐5683 expression in the Cancer Genome Atlas (TCGA) database. B, MiR‐5683 expression levels in 70 pairs of human GC tissues and adjacent normal tissues by miRNA RT‐PCR. C, MiR‐5683 expression in 70 pairs of human GC tissues and adjacent normal tissues. D, MiR‐5683 expression levels in GC cells and GES‐1. E, MiR‐5683 expression in cells transfected with lentiviruses expressing miR‐5683‐inhibitor and miR‐5683‐mimics. F and G, Proliferation of cells transfected with lentiviruses expressing miR‐5683‐mimics and miR‐5683‐inhibitor determined using a CCK‐8 assay. *p < .05, **p < .01, ***p < .001.

Firstly, we analyzed miR‐5683 expression in the TCGA database and found that miR‐5683 expression was obviously decreased in the GC specimens (Figure 1A). We then performed miRNA RT‐PCR in 70 paired GC and nontumorous tissues to determine the expression pattern of miR‐5683. The results indicated that miR‐5683 expression was severely decreased in GC tissues (Figure 1B,C). We also quantified miR‐5683 expression levels in normal gastric epithelial cell lines and GC cells lines. MiR‐5683 expression reduced obviously in GC cells (AGS, HGC27, BGC823, MGC803, and SGC7901) compared with GES‐1 (Figure 1D). Finally, we estimated associations between miR‐5683 level and clinical data from GC patients. GC patients were classified into low or high group based on their median miR‐5683 expression level. A negative association was found between miR‐5683 expression and tumor volume (Table 1).

**TABLE 1:** Expression of miRNA‐5683 expression and PDK4 in human gastric cancer according to patients' clinicopathological characteristics

Characteristics	Number	miRNA‐5683 expression		*P*‐value	PDK4 expression		*P*‐value
		High group	Low group		High group	Low group	
Age (y)							
<60	24	10	14	.314	14	10	.314
≥60	46	25	21		21	25	
Gender							
Male	44	20	24	.322	19	25	.138
Female	26	15	11		16	10	
Size (cm)							
<3	36	24	12	.004**	10	26	<.001***
≥3	34	11	23		25	9	
Histology grade							
Well‐moderately	28	13	15	.626	16	12	.329
Poorly signet	42	22	20		19	23	
Stage							
Ⅰ/Ⅱ	25	12	13	.803	14	11	.454
Ⅲ/Ⅳ	45	23	22		21	24	
T grade							
T1 + T2	27	13	14	.806	16	11	.220
T3 + T4	43	22	21		19	24	
Lymph node metastasis							
Present (N1‐N3)	47	27	20	.075	23	24	.799
Absent (N0)	23	8	15		12	11	

** *P* < .01, *** *P* < .001 statistically significant difference.

### 3.2 MiR‐5683 suppresses the proliferation of GC cells

**FIGURE 2:**
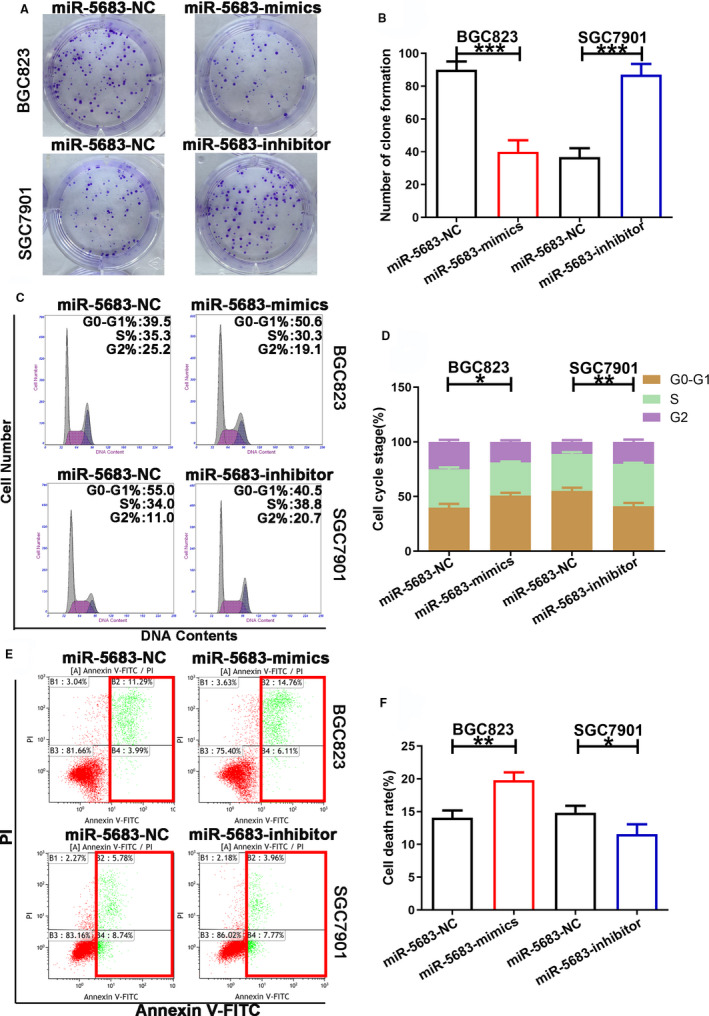
Effects of miR‐5683 expression on gastric cancer (GC) cell proliferation and apoptosis. A and B, Colony formation by cells transfected with lentiviruses expressing miR‐5683‐mimics and miR‐5683‐inhibitor. C and D, The effects of miR‐5683‐mimics or miR‐5683‐inhibitor on cell cycle distribution of GC cells. E and F, The effects of miR‐5683‐mimics or miR‐5683‐inhibitor on the cell apoptosis of GC cells. *p < .05, **p < .01, ***p < .001.

In order to uncover the biological effects of miR‐5683 in GC, lentiviruses expressing miR‐5683‐mimics were transfected respectively with BGC823 and AGS cells. Lentiviruses expressing miR‐5683‐inhibitor were transfected respectively with SGC7901 and HGC27 cells. The expression levels of miR‐5683 in GC cells were determined, and we verified the overexpression of miR‐5683 in BGC823 and AGS cells, and the downregulation of miR‐5683 in SGC7901 and HGC27 cells (Figure 1E; Figure S1A). Next, we carried out CCK‐8 assay to explore the effects of miR‐5683 on GC cell growth. The proliferation rate of BGC823 and AGS cells with miR‐5683 overexpression was markedly lower than that of control cells. However, miR‐5683 knockdown in SGC7901 and HGC27 cells increased proliferation rate (Figure 1F,G; Figure S1B,C). Similarly, in colony formation assay, miR‐5683 overexpression in BGC823 and AGS cells repressed GC cell proliferation, while miR‐5683 downregulation in SGC7901 and HGC27 cells produced opposite effect (Figure 2A,B; Figure S2A,B). MiR‐5683 overexpression in BGC823 and AGS cells was linked to a sharp enhancement in the fraction of cells during the G0/G1 period, while the contrary pattern was found in SGC7901 and HGC27 cells with miR‐5683 downregulation (Figure 2C,D; Figure S2C,D). Together, miR‐5683 overexpression is responsible for G0/G1 cell cycle arrest, while miR‐5683 downregulation produces the contrary effect.

### 3.3 MiR‐5683 induces gastric cancer cell apoptosis

Increasing apoptosis can inhibit the progression of tumors. Therefore, we applied flow cytometric analysis to detect cell apoptosis by Annexin V‐FITC staining in order to validate the assumption that miR‐5683 acts as a repressor in GC. The results revealed that miR‐5683 overexpression in BGC823 and AGS cells correlates with a higher rate of apoptosis. Nevertheless, miR‐5683 knockdown in SGC7901 and HGC27 cells produced the contrary effect (Figure 2E,F; Figure S2E,F). According to our findings, there is a positive relationship between miR‐5683 expression and cell apoptosis in GC.

### 3.4 PDK4 is a downstream target of miR‐5683

**FIGURE 3:**
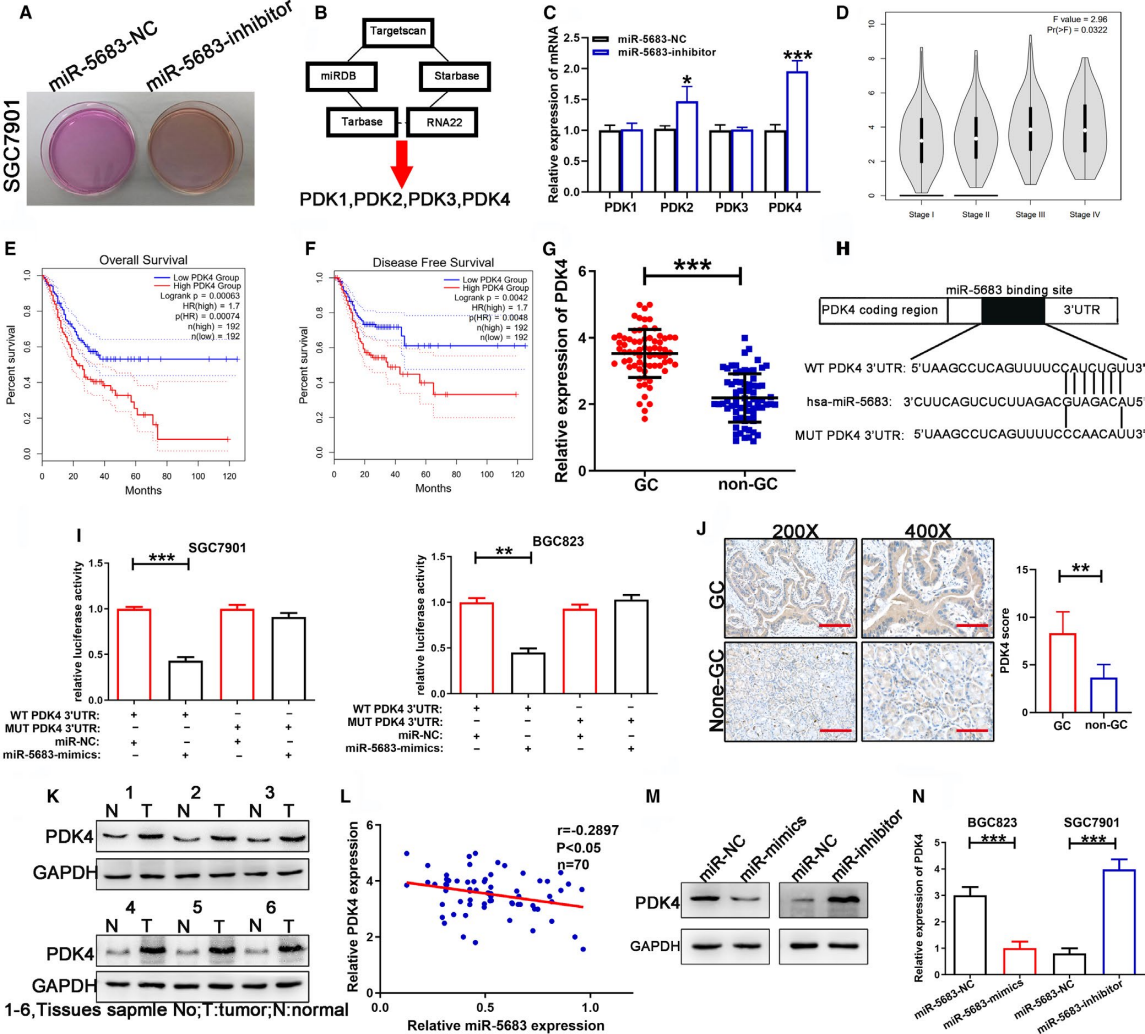
Pyruvate dehydrogenase kinase 4 (PDK4) was upregulated in gastric cancer (GC) tissues and cells, and PDK4 was identified as a direct target of miR‐5683. (A) The color of the culture medium in SGC7901. (B) Target gene searched on TargetScan, miRDB, starBase, Tarbase, and RNA22 prediction websites. (C) Relative expression of PDK1, PDK2, PDK3, and PDK4 after miR‐5683 downregulation. (D) The association of PDK4 with GC stage using GEPIA database. (E, F) Kaplan‐Meier analysis of the association of PDK4 with overall survival (E) or recurrence‐free survival (F) in patients with GC using GEPIA database. (G) PDK4 expression levels determined in 70 pairs of human GC tissues and adjacent normal tissues by qRT‐PCR. (H, I) The potential miR‐5683 binding site at the 3'‐UTR of PDK4 mRNA was computationally predicted by TargetScan. Luciferase activity was analyzed in cells co‐transfected with miR‐5683‐mimics or negative control and pGL3‐PDK4 or pGL3‐PDK4‐mut. (J) PDK4 protein expression in GC specimens and adjacent normal tissues determined by immunohistochemistry staining. (K) PDK4 protein expression was analyzed by western blotting in six pairs of GC tissues. (L) A negative correlation between the expression levels of miR‐5683 and PDK4 in GC specimens (*P* < .05). (M) PDK4 protein expression in GC cells transfected with lentiviruses expressing miR‐5683‐mimics and miR‐5683‐inhibitor. (N) Effects of changes in miR‐5683 expression on PDK4 mRNA expression in GC cells analyzed by qRT‐PCR. *p < .05, **p < .01, ***p < .001.

In the process of culturing GC cell lines, we discovered that in SGC7901 cells with miR‐5683 downregulation, the color of the culture medium altered from pink to orange more promptly (Figure 3A). We thus speculated that whether miR‐5683 downregulation could affect the glucose metabolism of GC, thus leading to GC progression. We then searched its target genes on the TargetScan, miRDB, starBase, Tarbase, and RNA22 prediction websites. We identified some glucose metabolism‐related genes (PDK1, PDK2, PDK3, PDK4) as potential targets for miR‐5683 (Figure 3B). Then we performed qRT‐PCR to determine the actual target of miR‐5683 among these predicted genes. Expression of PDK2 and PDK4 was significantly upregulated following a decrease in miR‐5683 (Figure 3C). In GEPIA database (http://gepia2.cancer‐pku.cn/#analysis), we found that the expression level of PDK4 was closely correlated with GC stage (Figure 3D). Moreover, higher PDK4 expression was associated with GC OS (HR = 1.7; *P* = .00074) and DFS (HR = 1.7; *P* = .0048) (Figure 3E,F). However, PDK2 showed no significant correlation with GC stage, OS, and DFS (Figure S3A‐C). We then analyzed PDK4 and PDK2 expression in 70 pairs of GC and corresponding nontumorous GC tissues. The results revealed the upregulation of PDK4 in GC tissues (Figure 3G), while only a slight upregulation of PDK2 was found in GC tissues (Figure S3D). Luciferase reporter assays were performed using cells transfected with pGL3‐PDK4 or pGL3‐PDK2 vectors with the wild‐type target sequence and cells transfected with pGL3‐PDK4‐mut or pGL3‐PDK2‐mut vectors containing the mutated 3'‐UTR target sequences. MiR‐5683 overexpression obviously reduced luciferase activity from pGL3‐PDK4 but not from pGL3‐PDK4‐mut, suggesting that miR‐5683 might bind to the PDK4 3'‐UTR (Figure 3H,I). However, the luciferase activity of the pGL3‐PDK2 reporter vectors displayed no change by ectopic expression of miR‐5683 (Figure S3E,F). By phosphorylating the E1α subunit of PDH, PDKs decrease its activity and reduce the conversion of pyruvate to acetyl‐CoA, resulting in the switching of carbon flow from the tricarboxylic acid (TCA) cycle and de novo lipogenesis to lactate production. We found that knockdown of miR‐5683 markedly increased the protein level of endogenous PDK4, leading to an increase in the phosphorylation of PDH‐E1α and to a marked reduction in PDH activity. However, knockdown of miR‐5683 slightly increased the protein level of endogenous PDK2. Knockdown of PDK4 in SGC7901 with miR‐5683 overexpression significantly decreased the phosphorylation of PDH‐E1α (Figure S2G). Based on the above results, we thus focused on the regulation of PDK4 by miR‐5683. IHC staining confirmed that PDK4 was overexpressed in GC tissues (Figure 3J). Western blot analysis also identified higher PDK4 expression in six GC tissues compared with corresponding nontumorous GC tissues (Figure 3K). A negative correlation was found between miR‐5683 and PDK4 expression with linear regression analysis (Figure 3L). The correlations between expression level of PDK4 and GC patients' clinical features also revealed a highly positive relationship between PDK4 expression and tumor size (Table 1).

### 3.5 MiR‐5683 suppresses expression level of PDK4 protein by mRNA degradation

The results of western blot showed that PDK4 protein level severely decreased in BGC823 cell line after miR‐5683 overexpression. On the contrary, the level of PDK4 protein increased in SGC7901 cell line with miR‐5683 knockdown (Figure 3M). Similar changes in PDK4 mRNA levels were also observed (Figure 3N). These findings suggested that miR‐5683 inhibits PDK4 expression via degradation of its mRNA.

### 3.6 PDK4 promotes the Warburg Effect in GC

To determine the functions of PDK4 in GC cellular metabolism, we knockdown PDK4 expression in SGC7901 cells and upregulated its expression in BGC823 cells by lentivirus infection. We found that knockdown of PDK4 reduced the phosphorylation of PDH‐E1α, thus increasing PDH activity. An opposite result was obtained after PDK4 overexpression (Figure S4A,B). PDK4 knockdown in SGC7901 cells markedly decreased glucose uptake and lactate production, whereas forced PDK4 expression in BGC823 cells had the opposite effects (Figure S4C‐F). ECAR and OCR, which reflect overall glycolytic flux and mitochondrial respiration, respectively, were then determined in GC cells. PDK4 knockdown in SGC7901 cells markedly decreased the ECAR rate, whereas PDK2 overexpression in BGC823 cells significantly enhanced ECAR (Figure S4G,H). The mitochondrial function of oxidative phosphorylation was also affected by PDK4 level. PDK4 knockdown in SGC7901 cells resulted in a markedly enhanced OCR relative to control cells. However, PDK4 overexpression in BGC823 cells led to lower OCR (Figure S4I,J). Taken together, these results show that PDK4 promotes the Warburg Effect in GC.

### 3.7 MiR‐5683 inhibits glycolysis via suppression of PDK4 expression in GC

**FIGURE 4:**
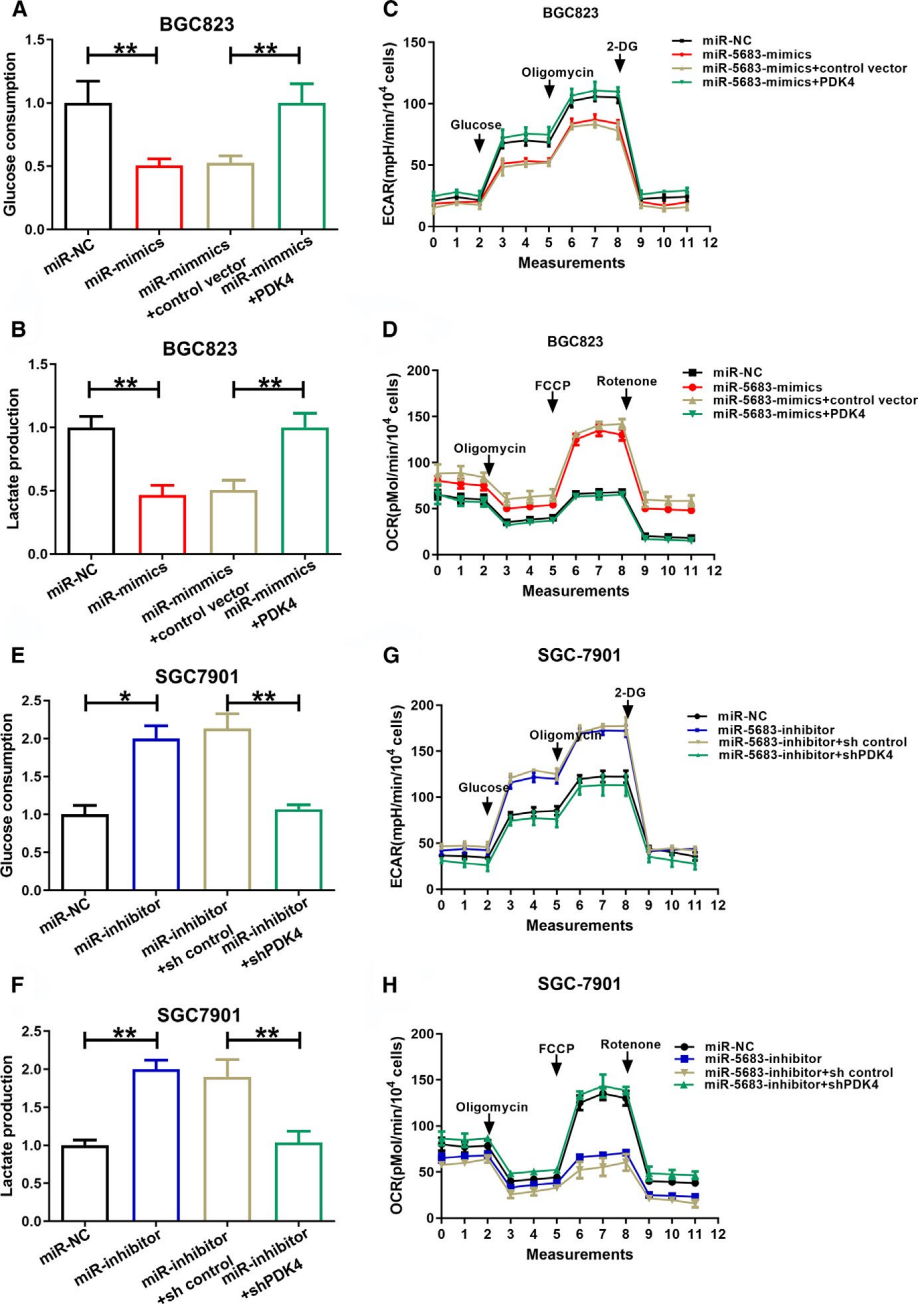
MiR‐5683 dampens glycolysis through inhibition of pyruvate dehydrogenase kinase 4 (PDK4) expression in gastric cancer (GC) cells. (A, B) Glucose uptake and lactate production by BGC823 cells transfected with lentiviruses expressing miR‐5683‐mimics or miR‐5683‐mimics plus the PDK4 expression vector. (C, D) Extracellular acidification rate (ECAR) and oxygen consumption rate (OCR) in BGC823 cells transfected as in (A) and (B). (E, F) Glucose uptake and lactate production by SGC7901 cells transfected with lentiviruses expressing miR‐5683‐inhibitor or miR‐5683‐inhibitor plus the shPDK4 vector. (G, H) ECAR and OCR in SGC7901 cells were transfected as in (E) and (F). All data are presented as the mean ± SD of quintuplicate measurements of three independent experiments. *p < .05, **p < .01, ***p < .001.

We then investigated whether PDK4 might mediate the influences of miR‐5683 on glycolysis in GC cells. BGC823 cells were transfected with miR‐5683‐mimics and either LV‐PDK4 (containing the PDK4 coding sequence without the miR‐5683 targeting sequence) or control vector. SGC7901 cells were transfected with miR‐5683‐inhibitor and either shPDK4 or shPDK4 control. MiR‐5683 overexpression in BGC823 cells reduced glucose uptake and lactate production (Figure 4A,B). ECAR and OCR were decreased and increased in BGC823 cells with miR‐5683 overexpression, respectively (Figure 4C,D). Such effects were reversed by PDK4 re‐expression (Figure 4A‐D). In contrast, miR‐5683 knockdown in SGC7901 cells increased glucose uptake and lactate production (Figure 4E,F). ECAR and OCR were increased and decreased in SGC7901 cells with miR‐5683 knockdown, respectively (Figure 4G,H). Those effects were rescued by PDK4 knockdown (Figure 4E‐H). The findings indicated that miR‐5683 dampens glycolysis by inhibiting PDK4 expression in GC cells.

### 3.8 MiR‐5683 regulates GC cell proliferation and apoptosis through targeting PDK4

**FIGURE 5:**
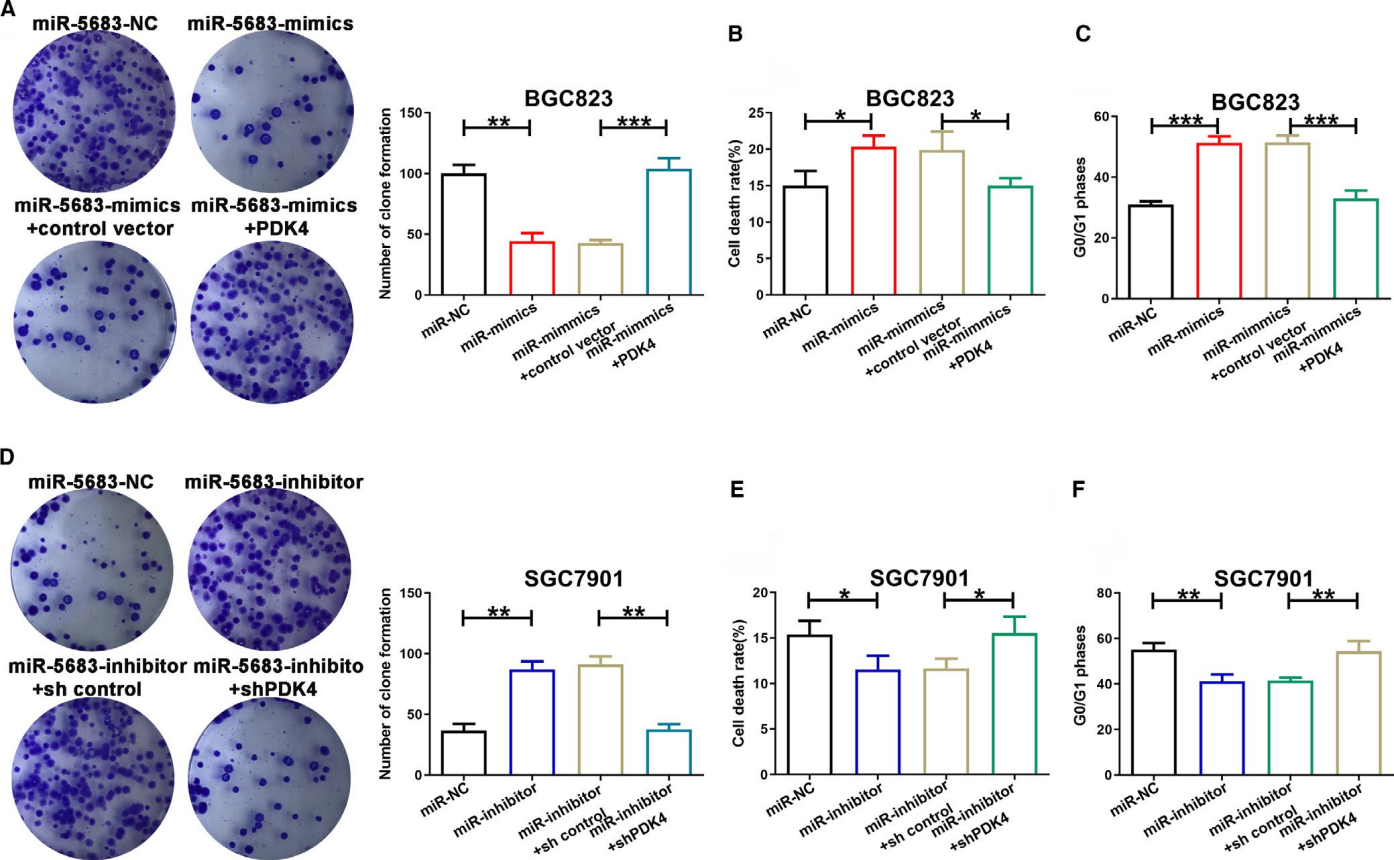
The roles of miR‐5683 and pyruvate dehydrogenase kinase 4 (PDK4) in the regulation of proliferation and apoptosis. A‐C, The reverse experiments for miR‐5683‐mimics were performed through the overexpression of PDK4. D‐F, The reverse experiments for miR‐5683‐inhibitor were performed through the downregulation of PDK4. Representative data are presented as mean ± SD values. *p < .05, **p < .01, ***p < .001.

Next, whether PDK4 could neutralize the phenotypes of miR‐5683 was explored. Results confirmed that miR‐5683 overexpression‐mediated repression of proliferation and promotion of apoptosis were neutralized by ectopic PDK4 expression (Figure 5A‐C). Intriguingly, miR‐5683‐inhibitor mediated similar effects. We noticed similar phenomenon in SGC7901 cell line where PDK4 was downregulated (Figure 5D‐F).

### 3.9 MiR‐5683 inhibits tumorigenicity in nude mice

**FIGURE 6:**
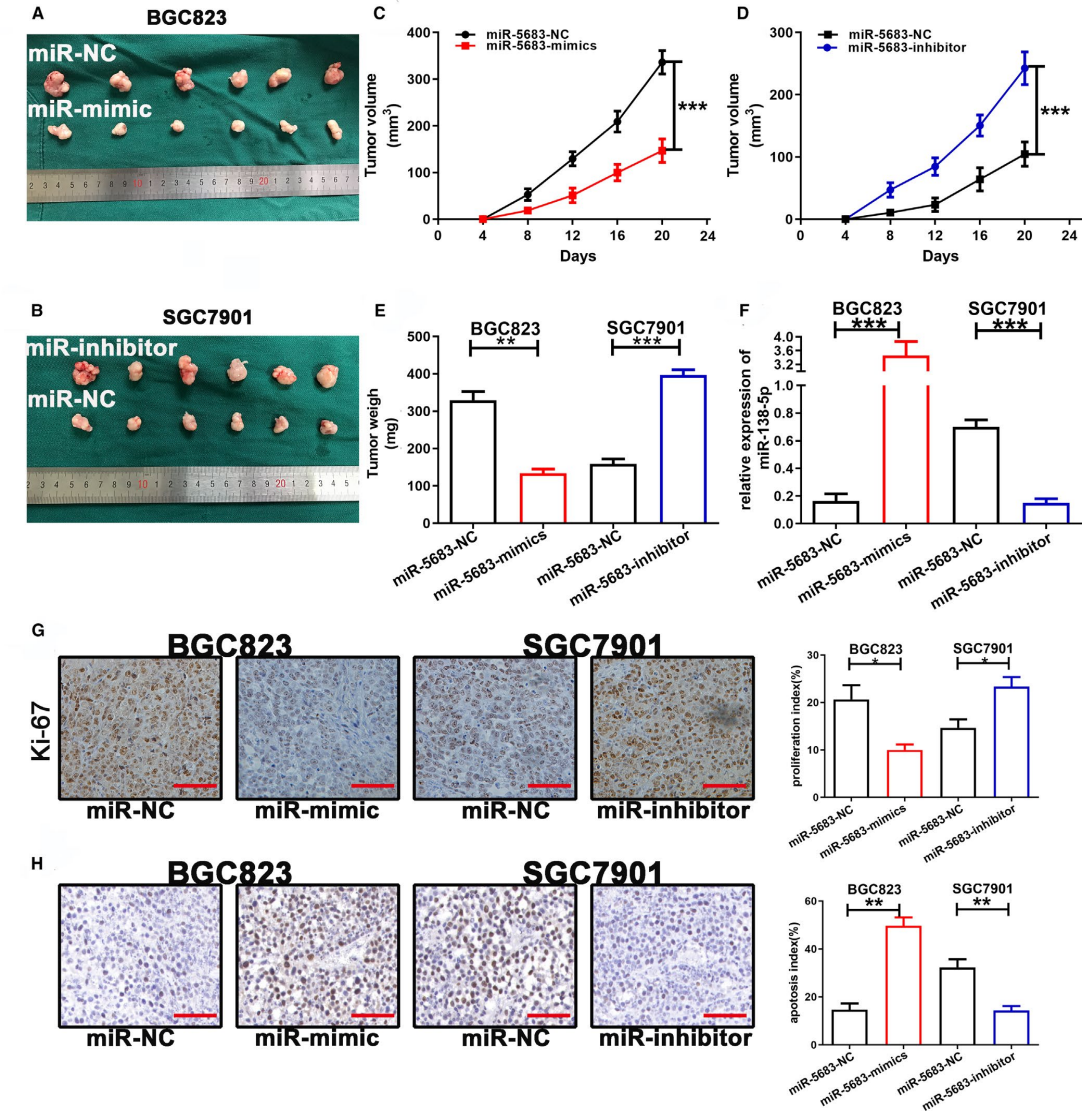
MiR‐5683 inhibited growth of gastric cancer cell xenografts in nude mice. A and B, Tumors were generated in female nude mice by subcutaneous injection of cells transfected with lentiviruses expressing miR‐5683‐mimics and miR‐5683‐inhibitor. C and D, Tumor growth (volume). E, Average tumor weight (*P < .05). F, MiR‐5683 expression in samples collected from nude mice. G and H, Immunohistochemical staining of Ki‐67 and TUNEL assay of the effects of changes in miR‐5683 expression on cell proliferation and apoptosis in the samples collected from nude mice. *p < .05, **p < .01, ***p < .001.

The influence of miR‐5683 on tumorigenicity was investigated using nude mice. At 20 days after injection of transfected cells, slowed tumor growth was observed in the miR‐5683‐mimics group compared with the corresponding control group (BGC823 cells). In contrast, accelerated tumor growth was observed in the miR‐5683‐inhibitor group compared with the corresponding control group (SGC7901 cells) (Figure 6A‐D). Mean weight of tumors in the BGC823 mimics group was obviously lower compared with the corresponding control group. It was also found that tumor weight was higher in the SGC7901 inhibitor group compared with the corresponding control group (Figure 6E). The expression of miR‐5683 was increased in the BGC823 mimics group, but downregulated in the SGC7901 inhibitor group (Figure 6F). Next, the ability of miR‐5683 to suppress tumorigenicity was evaluated by staining markers of proliferation (Ki‐67) and apoptosis (TUNEL). Compared to controls, the proliferation index in the BGC823 mimics group was reduced, but increased in the SGC7901 inhibitor group. Furthermore, in contrast to the control group, the apoptosis index was evidently higher and lower in the BGC823 mimics group and SGC7901 inhibitor group, respectively, (Figure 6G,H).

## 4 DISCUSSION

Gastric cancer is a frequently occurring malignant tumor at global scale and especially in China. While progress has been made, long‐term survival of GC patients remains poor.^16^ MiRNAs are short and noncoding and considered to regulate over half of all coding genes through binding to their mRNA, thus promoting their degradation or preventing their translation.^17^ Although recent study has shown that miR‐5683 promotes proliferation and the migratory abilities of colon adenocarcinoma cells, the expression pattern and effects of miR‐5683 on other types of tumors require further investigation.

In our study, we demonstrated a severe downregulation of miR‐5683 in TCGA, GC tissues, and GC cell lines. We also discovered that miR‐5683 inhibits proliferation and promotes apoptosis of GC cells. The opposite results were achieved through miR‐5683 knockdown. Hence, we conclude that miR‐5683 could suppress GC.

The mechanisms whereby miR‐5683 regulates GC proliferation and apoptosis remain unclear. The effects of miRNAs are attributable to the synergetic interaction of its targets because one miRNA can have hundreds of target genes and be involved in multiple biological processes. In our study, we show that miR‐5683 is involved in modulation of glucose metabolism. Through the color of the culture medium alteration and our searches using several computational algorithms, we focused on PDK2 and PDK4 as potential miR‐5683 targets. However, our experiments validated that PDK2 is not a direct target of miR‐5683. Upregulated PDK4 expression is associated with various cancers including acute myeloid leukemia, lung cancer, laryngocarcinoma, ovarian cancer, and colon cancer.^18^, ^19^, ^20^, ^21^ Overexpression of PDK4 also contributes to drug resistance, survival, and metastasis.^22^ A latest study that included the Asian cases has also shown that PDK4 functions as an oncogene in gastric cancer, which is consistent with our study.^23^ By phosphorylating the E1α subunit of PDH, PDKs decrease its activity and reduce the conversion of pyruvate to acetyl‐CoA, resulting in the switching of carbon flow from the TCA cycle and de novo lipogenesis to lactate production. In our study, we found knockdown of PDK4 reduced the phosphorylation of PDH‐E1α, thus increasing PDH activity. PDK4 knockdown decreased ECAR and increased OCR. We also demonstrated that miR‐5683 negatively controls PDK4 transcription by binding to its 3'‐UTR. Ectopic PDK4 expression can reverse the inhibition of proliferation and increase in apoptosis due to miR‐5683 upregulation. Besides, PDK4 shRNA rescued the effects of miR‐5683 knockdown. Hence, we argue that miR‐5683 inhibits tumorigenesis via a PDK4‐dependent mechanism.

Most cancer cells preferentially utilize glycolysis rather than mitochondrial oxidative phosphorylation (OXPHOS), even when oxygen is available. This metabolic reprogramming or Warburg effect^24^ requires a higher rate of glycolysis to meet increased macromolecular synthesis and rapid cancer cells proliferation,^25^, ^26^ and constitutes a valuable method for controlling tumor progression and strengthening antitumor effects.^27^, ^28^ PDK4 acts as a vital glucose metabolism‐related gene and is involved in the control of glucose metabolism and mitochondrial respiration.^21^, ^29^, ^30^ Thus, we wondered that whether PDK4 mediated the functions of miR‐5683 through regulating glycolysis. We revealed that miR‐5683 inhibits aerobic glycolysis by downregulating PDK4, demonstrating that miR‐5683 involves in regulating the PDK4‐mediated Warburg effect. A relevant consequence is that miR‐5683 upregulation can be effective in treating GC with PDK4 overexpression.

**FIGURE 7:**
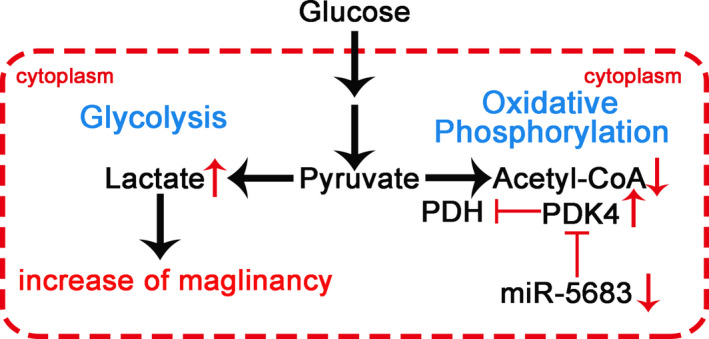
The schematic illustration of the mechanism by which miR‐5683 suppresses gastric cancer

In conclusion, we propose that miR‐5683 suppresses the progression of GC by targeting PDK4. We also demonstrate that the expression levels of miR‐5683 negatively relate to PDK4 protein levels in GC tissues, and are obviously reduced compared with corresponding normal tissues. The schematic illustration of how miR‐5683 suppresses GC is shown in Figure 7. The findings indicate the clinical significance of the tumor suppressor function of miR‐5683, and highlight miR‐5683 as a potentially novel therapeutic target in treating GC.

## CONFLICT OF INTEREST

All the authors declare no conflict of interest, financial or otherwise.

## AUTHOR CONTRIBUTIONS

ZX and YM designed and supervised the study. YM, QL, and GS performed all the experiments. LW performed data collection, sorting, and analysis. YM wrote the manuscript. DZ collected clinical tissue samples and analyzed patient information. HX provided technical support.

## Supporting information

Fig S1

Fig S2

Fig S3

Fig S4

## Data Availability

The analyzed datasets generated during the study are available from the corresponding author on reasonable request.
