# Supplementary figures and images for: MiR‐5683 suppresses glycolysis and proliferation through targeting pyruvate dehydrogenase kinase 4 in gastric cancer
Source: Cancer Med. 2020 Aug 11;9(19):7231–43. doi: 10.1002/cam4.3344 (PMC7541129; doi:10.1002/cam4.3344)

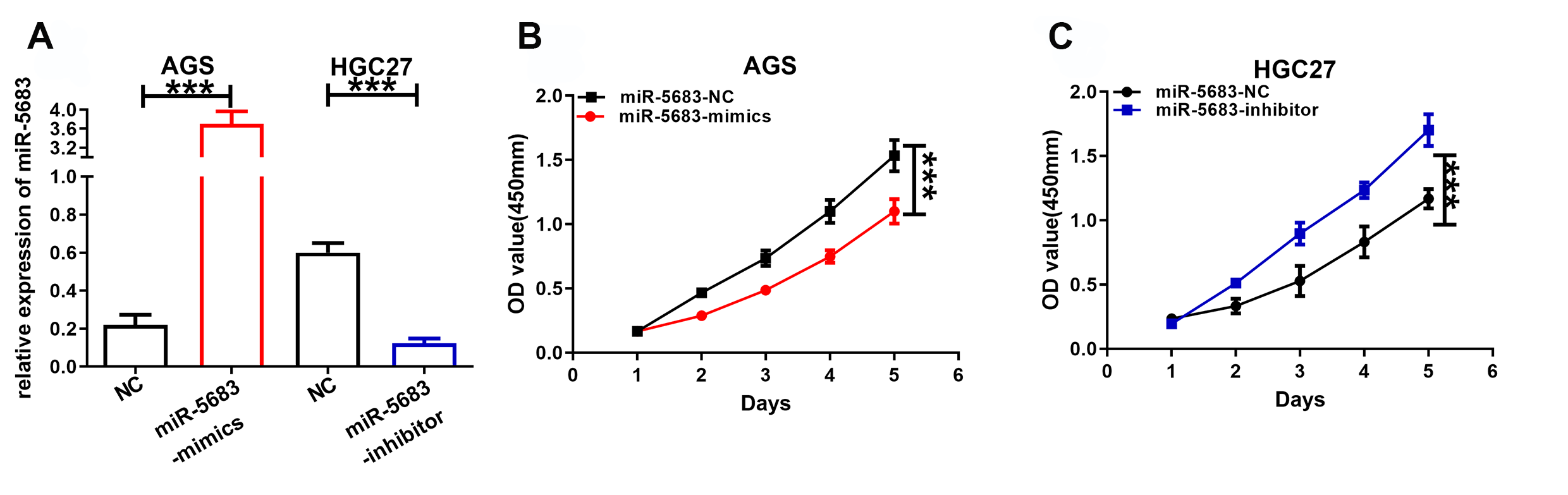

Supplement: Supplementary file 1 — Fig S1 [file CAM4-9-7231-s001.tif]

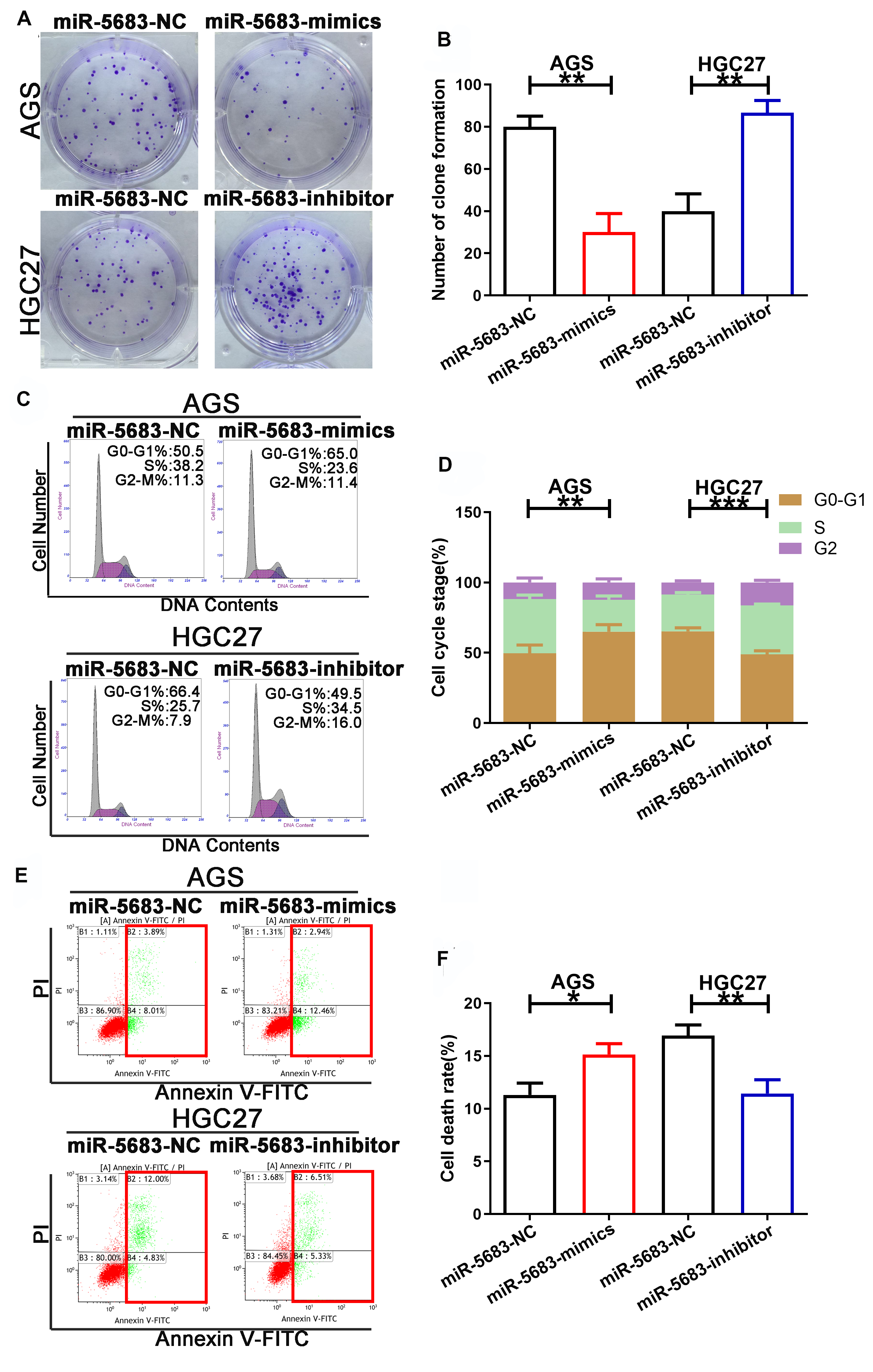

Supplement: Supplementary file 2 — Fig S2 [file CAM4-9-7231-s002.tif]

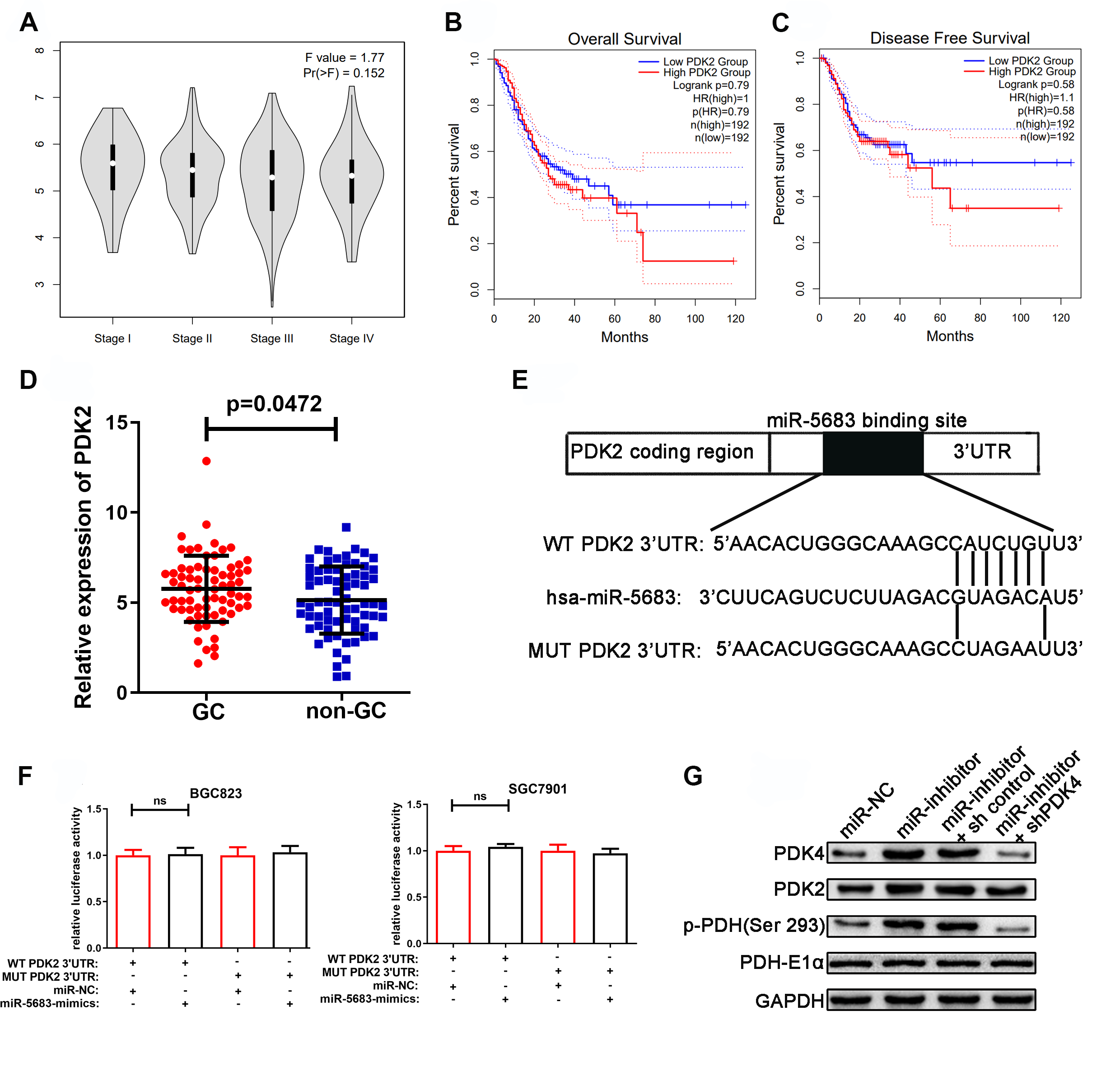

Supplement: Supplementary file 3 — Fig S3 [file CAM4-9-7231-s003.tif]

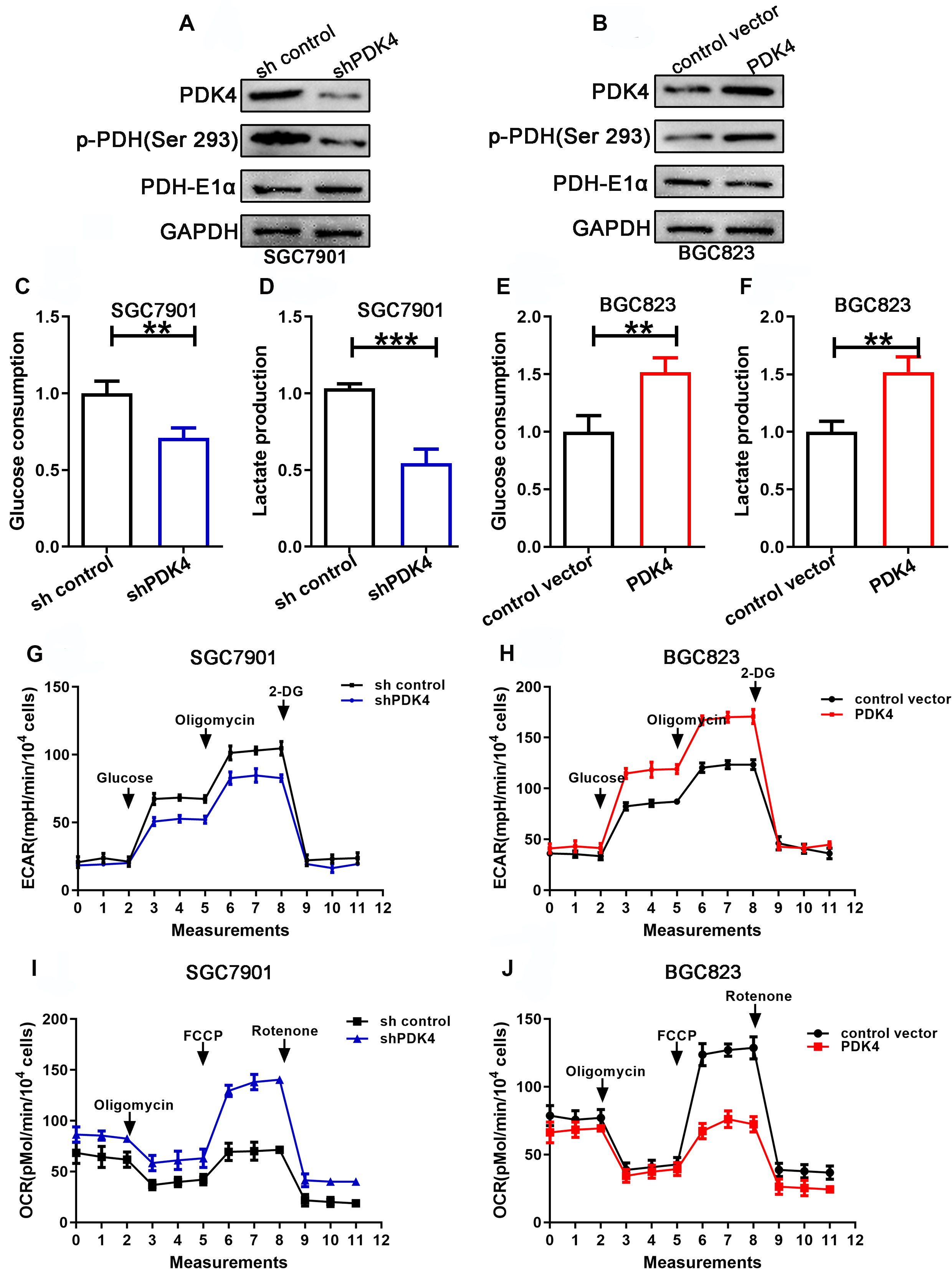

Supplement: Supplementary file 4 — Fig S4 [file CAM4-9-7231-s004.tif]
